# Organic Electrolytes Recycling From Spent Lithium‐Ion Batteries

**DOI:** 10.1002/gch2.202200050

**Published:** 2022-06-11

**Authors:** Ruihan Zhang, Xingyi Shi, Oladapo Christopher Esan, Liang An

**Affiliations:** ^1^ Department of Mechanical Engineering The Hong Kong Polytechnic University Hung Hom Kowloon Hong Kong SAR 999077 China

**Keywords:** battery recycling, carbon dioxide extraction, electrolyte recycling, global challenges, organic extraction

## Abstract

Lithium‐ion batteries (LIBs) are regarded to be the most promising electrochemical energy storage device for portable electronics as well as electrical vehicles. However, due to their limited‐service life, tons of spent LIBs are expected to be produced in the recent years. Suitable recycling technology is therefore becoming more and more important as improper treatment of spent LIBs, especially the aged organic electrolyte, can cause severe environmental pollution and threats to human health. The organic solvents and high concentration of lithium salts in aged electrolytes are always sensitive toward water and air, which would easily hydrolyze and decompose into toxic fluorine‐containing compounds, leading to severe fluorine pollution of the surrounding environment. Hence, recycling aged electrolytes from spent LIBs is an efficient way to avoid this potential risk to the environment. However, several issues inhibit the realization of electrolyte recycling, including the volatile, inflammable, and toxic nature of the electrolytes, the difficulty to extract electrolytes from the electrodes and separators, and various electrolyte compositions inside LIBs from different applications and companies. Herein, the current progress in recycling methods for aged electrolytes from spent LIBs is summarized and perspectives on future development of electrolyte recycling are presented.

## Graphical abstract

Aged electrolytes inside spent lithium‐ion batteries consist of volatile organic solvents and toxic lithium salts, which can cause severe environmental pollution and safety issues without proper treatment. This review summarizes the reported methods to recycle aged electrolytes as well as achieve progress during industrial recycling technology and proposes future perspectives for suitable electrolyte recycling directions.

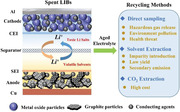

## 1 Introduction

With the increasing concerns of global energy crisis and climate changes, developing sustainable energy sources and realizing circular economy have become the main trends across the world in recent decades. Different kinds of renewable resources, including solar, biomass, hydro, wind, tides, waves and geothermal heat, have been explored as alternatives for the conventional fossil fuels to reduce the hazardous emissions and toxic pollution in our environment.^[^ ^1^, ^2^, ^3^ ^]^ However, electrochemical energy storage (EES) devices are always needed in the power generating system to efficiently transfer and use these renewable energies for other applications, such as portable electronics and electric vehicles (EVs).^[^ ^4^, ^5^ ^]^ Among the reported EES devices, lithium‐ion batteries (LIBs) have been regarded as the most promising choice due to their superior energy density, long cycling life, and high power output.^[^ ^6^, ^7^, ^8^, ^9^, ^10^, ^11^ ^]^ Specially, the number of LIBs for EVs increases dramatically during recent years with the acceleration of electrification in the transportation sector. The LIB energy storage capacity in the global market reached ≈218 GW h (over 1.2 million tons) in 2019, nine times as much as that in 2009 of nearly 25.6 GW h (≈134 000 tons), and the number is expected to increase to more than 2500 GW h (over 12.7 million tons).^[^ ^12^ ^]^ However, the average service lifespan of LIBs is only ≈3–5 years, which means that thousands of spent LIBs would be discarded in the near future.^[^ ^13^, ^14^ ^]^ As a typical solid electrical and electronic wastes, the spent LIBs always contain high content of valuable lithium (Li), nickel (Ni), and cobalt (Co) metals as well as some hazardous components including sensitive electrolytes and flammable additives.^[^ ^15^, ^16^ ^]^ The considerable amount of spent LIBs would consequently create significant environmental concerns, particularly as toxic heavy metals and harmful gases, if they are not adequately handled. On the other hand, tons of spent LIBs could be regarded as precious secondary resources of important raw materials for new LIBs production, from recovered graphite reused as anode, organic solvents for electrolyte, Li salts, and Ni/Co metal compounds for new cathode, and Al/Cu foil reused as current collectors.^[^ ^17^, ^18^ ^]^ Thus, it is urgent to develop suitable recycling technologies to obtain battery‐grade recovered raw materials and finally realize a circular LIBs supply chain.

**Figure 1:**
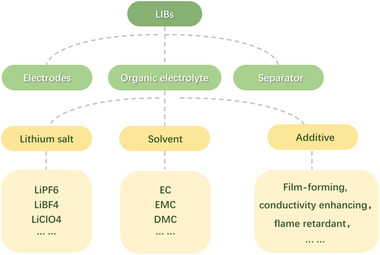
Illustration of organic electrolyte for LIBs.

**Figure 2:**
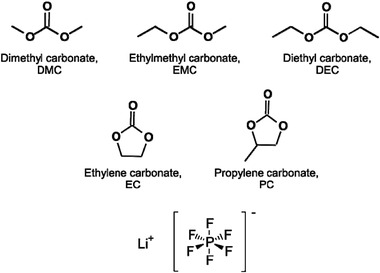
Structures of six of the most important organic carbonates and lithium salts LiPF_6_ for LIBs. Reproduced under the Creative Common Attribution License.^[^ ^47^ ^]^ Copyright 2017, The Authors, Published by MDPI.

**Figure 3:**
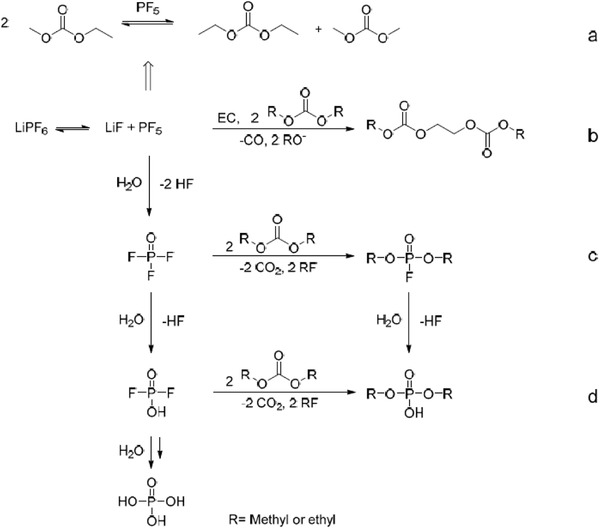
General decomposition pathways for the formation of: a) transesterifications products; b) oligocarbonate‐based products; c) organophosphate‐based products; and d) organic fluorophosphate‐based products and hydrolysis products. Reproduced under the Creative Common Attribution License.^[^ ^47^ ^]^ Copyright 2017, The Author(s), published by MDPI.

Tremendous efforts have been made to develop the efficient recycling technologies of spent LIBs. So far, there are three major recycling methods that have been fully studied, including hydrometallurgy, pyrometallurgy, and direct recycling methods, among which the first two processes have been applied in industrial scale while the last one is still in lab scale.^[^ ^19^, ^20^ ^]^ Due to high amount of valuable Li, Co, and Ni metals contained in cathode including lithium cobalt oxide (LiCoO_2_, LCO) and lithium nickel manganese cobalt oxide (LiNi_x_Co_y_Mn_1–_ *_x–y_* O_2_, NCM), all the recycling methods are mainly focused on the recovery of cathode materials in spent LIBs, especially the current industrial technologies.^[^ ^21^, ^22^, ^23^ ^]^ Other components, such as the graphite, binders, separators, organic electrolytes, and additives, are always burnt or abandoned in slag, which leads to huge emission of greenhouse gases and dust, causing serious environmental pollution. In particular, the organic electrolytes in spent LIBs easily react with air and water, causing severe secondary pollution and health threat toward human beings.^[^ ^24^, ^25^, ^26^, ^27^ ^]^ For commercially spent LIBs, the electrolyte consists of three parts (**Figure** **1**):^[^ ^28^ ^]^ the volatile carbonate solvents including methyl ethyl carbonate (EMC), ethylene carbonate (EC), and dimethyl carbonate (DMC) with different weight or volume ratios; toxic and sensitive lithium salts such as lithium hexafluorophosphate (LiPF_6_), lithium tetrafluoroborate (LiBF_4_), and lithium perchlorate (LiClO_4_); small amount of additives for film‐forming, conductivity enhancing, flame retardant, and overcharge protection.^[^ ^29^, ^30^, ^31^, ^32^ ^]^ The structures of the common solvents and lithium salt are shown in **Figure** **2**. During recycling process, the lithium salts would react with water or air to hydrolyze and decompose when they are exposed into environment, and finally some fluorine‐ and phosphorus‐containing compounds would be produced, which could lead to severe fluorine and phosphorus pollution.^[^ ^33^, ^34^ ^]^ At the same time, some reactions would occur for organic solvents, such as combustion, decomposition, and hydrolysis, causing the production of small organic alcohols (methanol and ethanol), aldehydes (formaldehyde and acetaldehyde), and acids (formic acid).^[^ ^14^, ^35^ ^]^ The typical reactions are described as follows (**Figure** **3**):

(1) $$\begin{array}{c} {\text{LiPF}}_{6}+H_{2}O\to 2\text{HF}+{\text{POF}}_{3}+\text{LiF} \end{array}$$

(2) $$\begin{array}{c} {\text{LiPF}}_{6}\to {\text{PF}}_{5}+\text{LiF} \end{array}$$

(3) $$\begin{array}{c} {\text{PF}}_{5}+H_{2}O\to 2\text{HF}+{\text{POF}}_{3} \end{array}$$

(4) $$\begin{array}{c} {\text{POF}}_{3}+H_{2}O\to \text{HF}+{\text{HPO}}_{2}F_{2} \end{array}$$

(5) $$\begin{array}{c} {\text{HPO}}_{2}F_{2}+H_{2}O\to \text{HF}+H_{2}{\text{PO}}_{3}F \end{array}$$

(6) $$\begin{array}{c} H_{2}{\text{PO}}_{3}F+H_{2}O\to \text{HF}+H_{3}{\text{PO}}_{4} \end{array}$$

(7) $$\begin{array}{c} {\left(C_{2}H_{5}O\right)}_{2}\text{CO}+H_{2}O\to {\text{CO}}_{2}+2C_{2}H_{5}\text{OH} \end{array}$$

(8) $$\begin{array}{c} {\left({\text{CH}}_{3}O\right)}_{2}\text{CO}+H_{2}O\to {\text{CO}}_{2}+2{\text{CH}}_{3}\text{OH} \end{array}$$

These produced substances from lithium salts and solvents are prone to dissolve and diffuse into water, soil, and air, which would result in severe environmental contamination and potential threats toward human life. Therefore, it is immeasurably terrible if the electrolytes in spent LIBs are not handled properly.^[^ ^36^ ^]^ Apart from the serious safety and environmental problems, the electrolyte that contains certain concentration of lithium salts plays an important role in the recovery of Li metal, which could make significant contribution to the green circular development in LIBs industries.^[^ ^37^, ^38^ ^]^

Although it is important to realize the recycling of electrolytes in spent LIBs, there are still some challenges for recycling technology development in LIB industry. As mentioned above, the electrolyte components exhibit special physical and chemical properties, especially the volatile, inflammable, toxic and sensitive nature, which increase the equipment cost and process complexity in recycling process design. Additionally, due to business confidentiality and growing competition among different companies, the components of electrolytes for LIBs on current market are varied and inconsistent, causing the time‐consuming pretreatment step during recycling.^[^ ^39^, ^40^ ^]^ What's worse, the aged electrolytes in spent LIBs are some kinds of liquids and most of them have been diffused and penetrated into the electrode structure during continuous cycling, which are hard to efficiently extract and collect.^[^ ^38^, ^41^ ^]^ In spite of the mentioned difficulties, several groups have been focused on the recycling of electrolytes from spent LIBs, and different methods have been developed to recover the organic solvents and lithium salts in the electrolytes, including solvent extraction,^[^ ^42^ ^]^ supercritical and liquid carbon dioxide (CO_2_) extraction.^[^ ^43^, ^44^, ^45^, ^46^, ^47^ ^]^ The comparison of the current recycling methods for aged organic electrolytes is presented in **Table** **1**. Furthermore, some industrial progress has been achieved by different companies, such as AEA Technology Batteries,^[^ ^42^ ^]^ OnTo Technology,^[^ ^48^ ^]^ and Accurec.^[^ ^49^ ^]^ In this review, we focus on the current status to the future perspective on the recycling of electrolytes in spent LIBs, which helps to appeal the global attention on toxic electrolyte and promote the overall sustainable development of the LIB recycling economy.

**Table 1:** Comparison of current recycling technologies toward aged organic electrolytes

Method	Yield	Dilution factor	Impurity	Organic waste	Products
Direct sampling	Low	–	Low	High	Only Li
Solvent extraction	Low	High	High	High	Li/Solvent
CO_2_ extraction	High	No	Low	Low	Li/Solvent

## 2 Current Status on Recycling Development on Aged Electrolytes in Spent LIBs

A typical LIB generally contains three main components: the graphite as anode, lithium, and transition metal compounds (LiFePO_4_ or LFP, LCO and NCM) as cathode, and an electrolyte containing high‐grade lithium salts (LiPF_6_ or LiBF_4_) dissolved in a dipolar organic solvent (EC, EMC or DMC). Generally, the anode and cathode materials are cast on copper and aluminum current collectors, respectively and then separated by a piece of organic separators (microporous polypropylene, PP). During assembling, proper amount of electrolyte solution is always injected into the battery to infiltrate the electrode materials and soak the separator, providing sufficient lithium ion for transfer during cycling. The weight ratio of every part in LIBs is listed in **Table** **2**,^[^ ^50^ ^]^ from which the electrolyte accounts for ≈10% weight of LIBs, almost equal to that of the current collectors. Thus, the electrolyte occupies a quite large portion of spent LIBs, and it is essential to consider the recycling of electrolyte into all recycling process in the long run. Besides, due to high concentration of lithium salts in electrolyte, the aged electrolyte is another important source for lithium recovery.

**Table 2:** The constructive components and their weight ratios in LIBs. Reproduced under the Creative Common Attribution License.^[^ ^50^ ^]^ Copyright 2019, The Authors, published by MDPI

Components	Weight ratio/w%	Most commonly used material
Case	≈25%	Steel/plastics
Cathode	≈27%	LCO, NCM, LFP
Anode	≈17%	Graphite
Current collectors	≈13%	Cu/Al
Electrolyte	≈10%	LiPF_6_ or LiBF_4_ dissolved in EC, EMC, DMC
Separator	≈4%	PP
Binder	≈4%	Polyvinylidene difluoride (PVDF)

**Figure 4:**
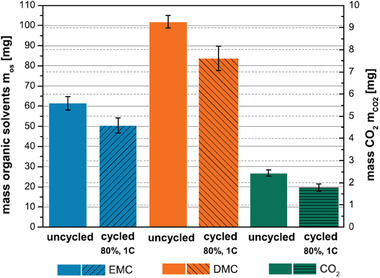
Preparation masses of the released gases EMC, DMC, and CO_2_ during crushing. Reproduced with permission.^[^ ^55^ ^]^ Copyright 2016. The Author(s), published by ECS.

In the spent LIBs, the aged electrolyte is always diffused into the whole electrode structure and even becomes immobilized after long‐term operation. With continuous cycling, the electrolyte is kept so as to form solid electrolyte interphases (SEI) on the electrode surface, leading to complicated side products in the electrolyte. Based on the complexity of composition and distribution, most research works and industrial recycling process usually focus on cathode recycling and therefore ignore the treatment details toward aged electrolyte, leading to immeasurable loss and hazardous emission toward our environment. In traditional recycling process, direct sampling method is generally used to treat the aged organic electrolyte, in which the aged electrolytes are directly removed by calcination or evaporation during pretreatment steps. Sumitomo and Sony in Japan developed a combined pyrometallurgical and hydrometallurgical processes, in which the plastics and electrolyte parts are directly burnt off during calcination at 1000 °C, leaving metallic parts and active materials for post treatment.^[38,^ ^39^ ^]^ The residual electrolytic solvents in spent LIBs can also be removed by evaporation at low temperature in an oven combined with the dry process of other parts.^[^ ^51^ ^]^ The lithium salts are always maintained in left active materials and recovered with other metal components by hydrometallurgical method.^[^ ^52^, ^53^, ^54^ ^]^ However, it has been proved that these thermal treatments during pretreatment steps always release toxic gases due to the decomposition of electrolyte. Diekmann et al.^[^ ^55^ ^]^ studied the released gas components during crushing step and found that the DMC, EMC, and CO_2_ were the main gases while the final ratio and types of released gases greatly depend on the solvents/lithium salts in electrolyte, and the state of health (SOH) of spent LIBs (**Figure** **4**). In addition, during dismantling step, the dominant released gases were found to turn into DMC and tert‐amylbenzene by Li et al.,^[^ ^56^ ^]^ and the emission quantities of these two gases reached 4.298 and 0.749 mg h^−1^, respectively, from one commercial 18650 cell. The findings mentioned above further confirm that improper treatment of aged electrolyte would cause huge waste and potential environmental risk in the long run. In contrast, recycling the aged electrolyte by solvent extraction, which can be divided into organic solvent extraction and supercritical extraction, can eliminate these problems.

### 2.1 Organic Solvent Extraction

**Figure 5:**
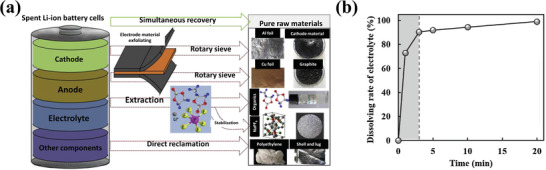
a) A schematic description of recycling process in this work. b) Dissolving rate of electrolyte solvent at the AEES as a function of time. Reproduced with permission.^[^ ^61^ ^]^ Copyright 2019, Elsevier B.V.

Extraction by organic solvent is one of the most promising methods for recycling organic electrolyte in spent LIBs.^[^ ^57^, ^58^ ^]^ The aged electrolyte is always dispersed into the pores of the electrode materials and separator. To extract the electrolyte, the electrode and separator parts are immersed in proper organic solvents, enabling the electrolyte to transfer into the organic solvent. After dissolving, the electrolyte is separated from the extraction solvents by distillation based on their different boiling point. Lain et al.^[^ ^42^ ^]^ from AEA Technology Batteries first applied several organic liquids to extract electrolyte from spent LIBs and found that the requirements for extraction solvents were that their boiling point at reduced pressure should be below the lithium salt decomposition temperature and the material should be available with an anhydrous state. However, there were too many impurities in the reclaimed electrolyte, making it impossible to reuse these electrolytes in new LIBs. Some carbonate solvents have also been applied to extract electrolyte from spent LIBs. The recovery efficiency of aged electrolyte with propylene carbonate (PC), diethyl carbonate (DEC), and 1,2‐dimethoxy ethane (DME) as extraction solvents was fully studied by Tong et al,^[^ ^59^ ^]^ and it was proved that PC could help to completely remove electrolyte within 2 h. Qiu et al.^[^ ^60^ ^]^ used EC as extraction solvent to recycle the electrolyte inside the cathode, anode, and separator of spent LIB. The reclaimed electrolyte was obtained after vacuum fractionation and exhibited good performance when reused in new LIBs. He et al.^[^ ^61^ ^]^ developed a green process for extracting aged electrolyte when exfoliating cathode and anode materials from spent LIBs (**Figure** **5**). They dissolved the organic solvents such as EC and PC into one aqueous exfoliating and extracting solution (AEES) and transferred LiPF_6_ salt into soluble NaPF_6_ and Li salt by reacting with AEES. With the help of AEES, almost all the electrolyte dispersed into the electrode materials and separator and could easily be dissolved within 20 min. These therefore show that all the electrolyte compounds could be easily obtained from AEES after distillation and filtration.

With organic solvent extraction, the aged electrolyte could be reclaimed from spent LIBs, which helped to increase the recycling value of spent LIBs and avoid the secondary pollution toward environment and human health caused by decomposition of electrolyte. However, the yield of organic solvent extraction is relatively low and large amount of organic solvents will be consumed, which might arise in new emission of organic waste from extraction solvents. In addition, the reclaimed electrolyte always contains little amount of organic impurities from extraction solvents, leading to the purification cost and low performance of reclaimed electrolyte when reused in new LIBs.

### 2.2 Supercritical Extraction

**Figure 6:**
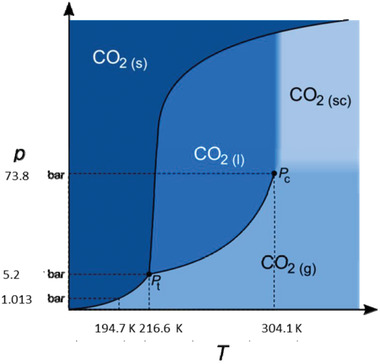
Pressure and temperature phase diagram of CO_2_. K: Kelvin, p: pressure, T: temperature, sc: supercritical, l: liquid, g: gaseous, s: solid. Reproduced under the Creative Common Attribution License.^[^ ^47^ ^]^ Copyright 2017, The Author(s) published by MDPI.

Supercritical phase is described as a state of substance between gas phase and liquid phase and supercritical fluids always possess gas‐like viscosity and liquid‐like density, which could easily diffuse into solid matrix that is hard compared to liquids. When it is close to the critical point, the properties of supercritical fluids could be fast changed by varying the surrounding pressure and temperature, enabling different substances in supercritical fluid to be extracted and separated based on their different solubility.^[^ ^62^, ^63^ ^]^ Among different supercritical liquids, CO_2_ is a desirable extraction medium for electrolyte recycling in spent LIBs due to its easily achievable critical point at 7.38 bar pressure and 31.1 °C, large dissolvability of non‐polar organic solvents, mature extraction and separation technology, and being non‐toxic and pollution‐free in reclaimed electrolyte (**Figure** **6**).^[^ ^64^, ^65^ ^]^

**Figure 7:**
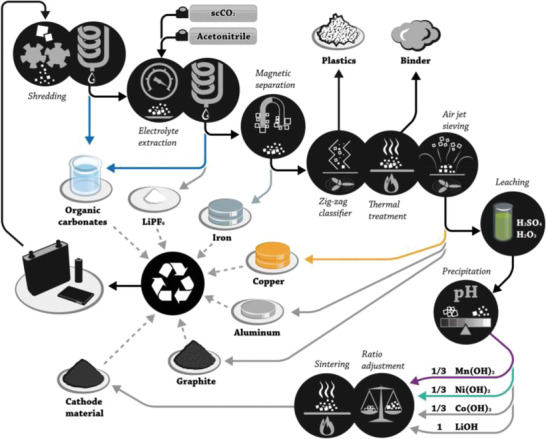
Flowsheet for the LithoRec battery recycling process. Reproduced with permission.^[^ ^66^ ^]^ Copyright 2016, Wiley–VCH.

Many works have been reported on the supercritical CO_2_ extraction for LIB electrolytes. Nowak et al.^[^ ^43^ ^]^ first applied the supercritical CO_2_ extraction with helium head pressure on electrolyte recycling from spent LIBs through a static autoclave setup. In this work, the electrolytes of commercial 18650 cells after formation and aging steps were extracted, containing the solvents of EC, DMC, and EMC, which were identified by gas chromatography (GC). Furthermore, the electrolyte aging products were also determined via different ionization modes in GC–mass spectrometry (GC–MS) experiments, and four electrolyte degradation products formed during cycling were successfully detected and extracted, which were confirmed as dimethyl‐2,5‐dioxahexane dicarboxylate (DMDOHC), DEC, diethyl‐2,5‐dioxahexane dicarboxylate (DEDOHC), and ethylmethyl‐2,5‐dioxahexane dicarboxylate (EMDOHC). The concentration of these four aging products was associated with the aging temperature and SEI growth. However, the recovery amount of lithium salts (LiPF_6_) was very low, and the total extraction rate was slow. In order to improve the recovery rates and reduce the extraction times, they added some different solvents into supercritical and liquid CO_2_ (sc and liq CO_2_) extraction, namely a flow‐through method.^[^ ^45^ ^]^ It was found that the recovery rate of cyclic EC was higher in sc CO_2_, while the linear carbonates such as DMC and EMC were recovered more in liq CO_2_. Besides, the addition of solvents including acetonitrile (ACN), DEC, and PC is also found to enable improved recovery rates for all the components after adding into CO_2_, especially for LiPF_6_. With the addition of ACN/PC solvents in a ratio of 3:1 into liq CO_2_ at 25 °C and 60 bar, nearly (89.1 ± 3.4) wt% electrolyte in commercial 18650 cell could be recovered. In addition, the detection of aging products like DEDOHC confirmed that the CO_2_ extraction could act as an effective tool to predict the aging and postmortem of electrolyte in LIBs. To verify the feasibility of graphite anode recycling coupled with electrolyte extraction, they compared three different electrolyte extraction concepts including thermal drying without the electrolyte recovery, flow‐through supercritical CO_2_ extraction with ACN addictive, and static supercritical CO_2_ extraction without co‐solvents (**Figure** **7**).^[^ ^66^ ^]^ It was verified that ACN assisted flow‐through supercritical CO_2_ extraction exhibited the highest recovery efficiency of electrolyte (90%) and the obtained recovered graphite displayed comparable performance to commercial ones after subsequent thermal treatment in this recycling procedure. The same group further used the supercritical CO_2_ extraction method combined with GC‐electron impact (EI) ionization and mass selective detection to figure out the influence of electrolyte aging on performance fading of LIBs during long‐term cycling.^[^ ^46^ ^]^ With the help of supercritical CO_2_ extraction, 17 aging products of electrolyte were confirmed and analyzed, proving this combined method to be an efficient way to detect electrolyte decomposition inside LIBs.

**Figure 8:**
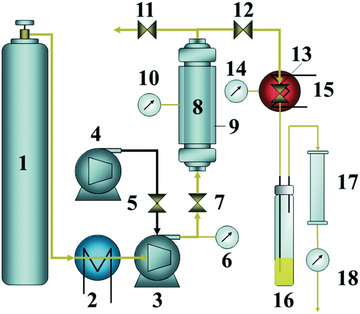
Schematic diagram of supercritical CO_2_ extraction apparatus: 1) CO_2_ cylinder, 2) cooling bath, 3) air driven fluid pump (gas booster pump), 4) air compressor, 5) air regulator, 6) CO_2_ pressure, 7) inlet valve, 8) extraction vessel, 9) heating jacket, 10) vessel heat, 11) vent valve, 12) outlet valve, 13) flow valve, 14) valve heat, 15) heating jacket, 16) collecting vial, 17) alumina filter, 18) gas flow meter. Reproduced with permission.^[^ ^44^ ^]^ Copyright 2014, Royal Society of Chemistry.

**Figure 9:**
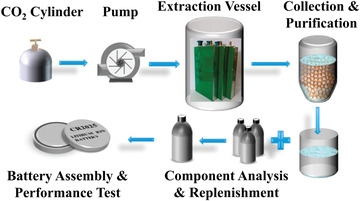
Schematic of reclaimed electrolytes from spent LIBs. Reproduced with permission.^[^ ^68^ ^]^ Copyright 2017, American Chemical Society.

Dai et al. also focused on the application of supercritical CO_2_ extraction for recycling electrolyte from spent LIBs.^[^ ^44^, ^67^, ^68^ ^]^ They first optimized the operational conditions of supercritical CO_2_ extraction by adopting a 3D response surface methodology (as shown in **Figure** **8**).^[^ ^44^ ^]^ It was confirmed that the extraction pressure played an important role in the recovery rate of organic carbonate solvents via supercritical CO_2_ extraction, while the LiPF_6_ was hard to be recovered and it hydrolyzed in supercritical CO_2_ extraction. The condition parameters for supercritical CO_2_ extraction were examined with pressure of ≈15–35 MPa, temperature of ≈40–50 °C and static extraction within ≈45–75 min. The highest extraction yield (85.07 ± 0.36%) was obtained at 23 MPa, 40 °C after dynamically extracting for 45 min. Afterward, they further analyzed the relationship between the physical properties of electrolyte components and their extraction behavior in supercritical CO_2_ under different pressure and temperature conditions.^[^ ^67^ ^]^ Based on their results, it was concluded that the polarity and melting point of carbonate were the controlling factors toward the extraction behavior of EC, DMC, and EMC in supercritical CO_2_, which would deeply affect their separation and fractionation rates. In addition, they further present a novel approach for the recycling electrolyte from spent LIBs, which is by combining supercritical CO_2_ extraction with purification by resin deacidification and molecular sieve dehydration as well as components supplement (as shown in **Figure** **9**).^[^ ^68^ ^]^ It is demonstrated that, four different carbonate solvents including vinylene carbonate (VC), EMC, DEC, and EC were successfully extracted and a high recovery rate was obtained for linear carbonates (EMC and DEC). With this novel recycling approach, the reclaimed electrolyte displayed a comparable ionic conductivity of 0.19 mS cm^−1^ to that of a commercial electrolyte with the same composition and exhibited superior electrochemical stability at 5.4 V (vs Li/Li^+^). When reused in Li/LCO cell, the reclaimed electrolyte enabled the cell to present an initial capacity of 115 mAh g^−1^ and high‐capacity retention rate of 66% after 100 cycles under 0.2 C.

Although great progress has been achieved on supercritical CO_2_ extraction for electrolyte recycling in lab scale, some critical issues still inhibit the wide application of this extraction method in industry scale, especially its intrinsic high cost.

### 2.3 Other Progress in Electrolyte Recycling in Laboratory and Industry

Apart from the extraction method, Zhong et al.^[^ ^67^ ^]^ developed a recycling process which combined pyrolysis and physical separation to recover the components in spent LFP‐based LIBs. In this novel process, the organic electrolyte was reclaimed via low‐temperature volatilization at 120 °C in a flat glass tube under high nitrogen protection, while, the LiPF_6_ salt was disposed by pyrolysis process at 550 °C to form HF and H_3_PO_4_ in water at above 180 °C under nitrogen. The results of low temperature volatilization in this work showed that ≈99.91% of the organic electrolytes was recovered for 50 min. Besides, the content of harmful elemental fluoride was only 0.067% after the pyrolysis, indicating that the treatment of LiPF_6_ in this recycling process was harmless.

Currently, some companies have tried to realize the partially or even full recovery of electrolyte from spent LIBs coupled with electrode recycling process. The AEA Technology Batteries extract the electrolyte with suitable organic liquid solvents and finally collect the pure electrolyte by evaporation at reduced pressure after being separated from solid residuals.^[^ ^42^ ^]^ Similarly, the OnTo Technology realizes electrolyte recycling by extraction method but using supercritical CO_2_ instead of organic solvents.^[^ ^48^ ^]^ By circulating CO_2_ flow or immersing spent LIBs into supercritical fluid, nearly 90% of the electrolyte is successfully extracted after 48 h. The IME develops a combination of pyro‐ and hydro‐metallurgical method, and collects the electrolyte through evaporation and condensation during pretreatment process.^[^ ^38^ ^]^ The condensation technology is also applied to recover electrolyte in the Accurec recycling process.^[^ ^49^ ^]^ Differently, the electrolyte is collected via a vacuum pyrolysis treatment under 250 °C instead of evaporation before condensation. Although some companies have paid close attention to electrolyte recycling, more attention should be directed to electrolyte recycling not only on lab‐scale but also on industrial‐scale to curtail the growing environmental pollution and health threat in the long run. It is thus urgent to realize the full recycling of every component in LIB and turn them to a truly green energy technology.

## 3 Future Perspectives on Electrolyte Recycling From Spent LIBs

As one of the basic parts in LIBs, electrolyte plays important roles in ensuring high lithium‐ion conductivity and maintaining good electrochemical and thermal stability during cycling. Three components including dipolar aprotic mixture of carbonates, high grade lithium salts, and trace of additives make the electrolyte with the features of volatility, inflammability, toxicity, and sensitivity toward water and air. Considering the different requirements of LIBs in different application condition, the composition of electrolyte varies a lot.^[^ ^40^, ^69^ ^]^ Furthermore, due to growing competition among different companies, the electrolytes become more complicated, exacerbating the difficulty of efficient electrolyte recycling. However, the aged electrolytes in spent LIBs exhibit huge threat to environmental safety and human health if they are not properly handled and treated. Owing to the high fraction in the overall weight of LIBs, the aged electrolytes hold attractive economic benefits, especially for Li recovery.^[^ ^70^, ^71^ ^]^ Although the recycling of spent LIBs has been widely studied and substantial progress has been made toward electrode and current collector recycling, the attention and interest on electrolyte recycling is still far away from enough at both research and industrial level. Considering the environmental risk from the aged electrolyte, realizing sustainable and efficient recycling would play pivotal role in making LIB a truly green energy source and finally addresses the energy crisis and climate change across the world. With these considerations, perspectives on future research directions on electrolyte recycling pertaining to LIBs are as follows:

1) Design safe and green electrolyte for LIBs or other suitable EES devices. Currently, the dominant electrolyte for LIBs is composed of aprotic solvents and sensitive lithium salts with proper additives. Other kinds of electrolyte, such as aqueous solution,^[^ ^72^, ^73^ ^]^ ionic liquid,^[^ ^74^, ^75^ ^]^ and solid state electrolyte,^[^ ^76^, ^77^, ^78^ ^]^ exhibit more promising properties than the organic ones, including non‐toxic, low cost, stability, and safety. Designing safe and green electrolyte is thus the best way to replace the organic electrolyte.
2) Optimize the internal structural design of LIBs to ease the recycling of all parts in spent LIBs. The current structure design of LIBs makes it difficult to efficiently separate and dismantle the components, especially the electrolyte part, in which the electrode materials have to be immersed and soak the separator. If the structure of LIBs could be further optimized to easily separate every component, the recycling of spent LIBs would be more efficient and easily achieved.
3) More attention is required on the electrolyte recycling from current spent LIBs. Although the extraction method has been proven to be effective on electrolyte recycling, the extraction solvents should be eco‐friendly and affordable with mature production technology. The recycling process should be designed from systematic perspective, which could realize the electrode recovery and simultaneously enable the electrolyte recycling. The typical organic electrolytes in LIBs comprise three parts: organic solvents, lithium salts, and additives. Currently, after recycling, the main recovered products from the aged electrolytes are the lithium‐based composites and the organic solvents, where the later can only be recovered by extraction methods. Therefore, from the recovered products perspective, it is proposed that the future electrolyte recycling methods can be divided into two steps: the first step is to recover the organic solvents from spent LIBs and the second step is to reproduce the lithium salts separately or combine with other metal products. It is believed that, through using such a two‐step method for future electrolyte recycling, the recycled lithium salts from the electrolyte could effectively relieve the tight global supply of lithium metal.
4) Establishment of a reliable and consistent business model from producer to consumer to recycler is necessary. The producer should provide similar or consistent electrolyte composition design toward one special application and also ensure that the electrolyte in new LIBs are easily distinguished and classified, thereby reducing the composition complexity from the beginning of LIB production. Besides, every producer should be responsible for the recycling of their own LIBs. They could encourage the consumers to return the spent LIBs by paid acquisition and collect the spent LIBs to be recycled with the established industrial technology. The consumers should also pay attention to the proper treatment of the spent LIBs, especially on the toxic aged electrolyte. They can make contribution to the classification of spent LIBs and be involved in essential supervision of producers and recyclers. The recyclers should efficiently sort and collect the spent LIBs and treat the aged electrolyte with proper methods following the local and standard procedures. The recyclers could also provide insightful suggestions for the producers toward sustainable electrolyte design.

## Conflict of Interest

The authors declare no conflict of interest.
